# The Demand for Disaster Microinsurance for Small Businesses in Urban Slums: The Results of Surveys in Three Indian Cities

**DOI:** 10.1371/currents.dis.83315629ac7cae7e2c4f78c589a3ce1c

**Published:** 2017-03-01

**Authors:** Ronak Patel, Garrett Walker, Mihir Bhatt, Vishal Pathak

**Affiliations:** Stanford University; Independent Researcher, Los Altos, California, USA; All India Disaster Mitigation Institute; All India Disaster Mitigation Institute

## Abstract

**Background:**

Small informal businesses make up the core markets for many poor urban communities, providing essential goods, services, and livelihoods. Many of these communities and businesses exist in hazardous locations. In most cases, these business owners do not have access to proper coping mechanisms including risk transfer and lack resilience to shocks. Access to risk-transfer in the form of insurance for these small businesses is extremely limited. This demand survey is the first phase of an intervention to test disaster microinsurance for these businesses. Previous research has examined the demand for and value of microinsurance to protect poor households but not micro- and medium-sized informal urban businesses.

**Objective:**

This study investigates knowledge about and demand for microinsurance among small informal business owners in three different cities of India.

**Methods:**

Survey of all informal business owners (n=4919) identified through purposive sampling of the most vulnerable in three proposed study sites: Guwahati in Assam (n=1622), Puri in Odisha (n=1551) and Cuddalore in Tamil Nadu (n=1746).

**Results:**

Our findings reflect that while small business owners largely did not know about disaster microinsurance, after describing it, a vast majority wanted to subscribe to such a program. Without it, they often rely on personal savings, forgo basic necessities, or take out costly loans that trap them in debt to cope with disasters.

**Discussion:**

This research supports the need for more experiments on actual adoption patterns, feasibility studies, and innovative trial programs by governments, non-governmental organizations, and insurance providers.

## Introduction

The 2015-2016 Slum Almanac published by UN-Habitat showed the dramatic extent of urban poverty: over one billion people currently live in slums.^1^ To combat this problem, the UN’s newly agreed Sustainable Development Goals (SDGs) include a focus on cities — a call for improving slums and creating more sustainable communities. SDG Number 11 calls for making cities safe, inclusive, resilient, and sustainable.^2^ However, natural and man-made disasters hinder progress made toward these goals.

Furthermore, humanitarian efforts tend to focus on the response to crisis rather than the long-term goal of improving resilience. The most recent World Humanitarian Summit highlighted the need to place humanitarian response within a broader development and resilience framework.^3^ By embracing a focus on resilience, development and humanitarian efforts can work together in complementary ways. Working towards resilience involves not just making infrastructure more robust, but also instituting risk mitigation techniques designed to promote appropriate investment and safeguard progress in the wake of disasters. These incremental steps improve the stability of a community and often do more good than reactive measures following catastrophes.^4^ However, while more groups are looking at how humanitarian aid can better adapt to urban crises — specifically those affecting informal settlements — few effective tools exist.^5^

In many cases, small and informal businesses provide livelihoods and offer goods and services to urban communities. Because of this, small-scale and decentralized market enterprise may be one of the best ways to identify and address needs in urban settings. People living in slums rely on small and informal businesses for inexpensive, reliable goods that satisfy their immediate needs and make other commerce possible. As a result, failure of these businesses represents failure of local markets in these communities.

Since local markets fulfill such a necessary role, humanitarian efforts have looked to salvage businesses directly after disasters. Cash programming previously showed promise; supporters hoped to spur demand in local markets and provide capital for businesses to operate.^6^^,^ 7 Unfortunately, small businesses feel the effects of disasters too, preventing or limiting recovery after a shock. And even when businesses can operate, introducing cash into a disaster-affected community can inflate demand without increasing supply. With limited supply, and little outside competition, merchants can simply raise their prices, adding to local inequality and risking high inflation.^8^ Even in open-market communities, cash assistance alone is not a complete answer; it fails to support local markets and businesses that are equally affected by the crisis.

Disaster microinsurance may be part of the answer. Several previous studies have proven the efficacy and reliability of microinsurance.^9^ Disaster microinsurance, which uses smaller premiums to cover smaller losses, has specifically been tested in endangered low-income households.^10^ The concept of microinsurance is simple: When businesses are doing well, they pay insurance companies a relatively small amount against the risk of natural disasters or significant environmental pressures. Should calamity strike, insurers compensate the business owners — potentially saving their businesses, their livelihoods, and their families. In doing so, the insurance industry transfers risk away from small businesses, which play an important role in local economies. Through this process, microinsurance provides an important link between humanitarian and development assistance.

Yet research into the impacts of disaster microinsurance has been limited. Some studies have shown that microinsurance has helped people recover from disasters faster than other coping strategies.^11^ And insurance companies have voiced their willingness to create programs. In 2005, insurance providers in Bangladesh, India, Pakistan, and Malawi all expressed interest in marketing their products to the poor. On a global scale, creative alliances between NGOs, insurance companies, governments, and donor organizations could enable microinsurance programs to be successful.^12^ But assumptions that informal business owners are uninterested, questions about financial feasibility, logistical challenges, and insufficient knowledge of the actual impacts understandably contribute to a lack of microinsurance programs in urban markets.

Additionally, questions about the merits of microinsurance in these settings remain. Some argue that microinsurance may create a form of moral hazard, actually promoting unsustainable or dangerous endeavors.^13^ Furthermore, since microinsurance has not been rolled out on a large scale, adoption rates — and, subsequently, financial viability — cannot yet be determined. The premiums may be too high to afford, business owners may lack understanding of microinsurance, or business owners without extensive financial experience may be wary of financial programs — a recurring issue due in part to prior scams.

Indian cities are an ideal setting to test microinsurance as a tool for addressing urban poverty and increasing community resilience. In the fastest-growing urban areas, over half of the population lives in slums. In the financial hub of Mumbai, for example, slums house roughly 54% of urban residents.^14^ Many dense urban slums struggle with natural disasters, including storms and floods — exactly the kind of unpredictable but expected hazards that microinsurance programs are designed to protect against.

Specifically, Southeast India is disproportionately affected. Due to its location in one of the seven major cyclone basins, the region frequently struggles with severe storms. Since 2010, for example, Cyclones Laila, Jal, Thane, Phailin, Hudhud, and Nada, and Vardah have all hit the southeast coast of India.^15^ And floods — the most common natural disaster in India — endanger the entire coast of India.^16^ All of the study sites — Puri in Odisha, Guwahati in Assam, and Cuddalore in Tamil Nadu — are located in this volatile region.

Between 1990 and 2016, the total loss from natural disasters in India has been assessed at over 48 billion US dollars.^17^ The insurance sector compensated just 11-12% of that amount, revealing an enormous gap in coverage and an opportunity for microinsurance.^18^ Additionally, informal employment contributes 48% of India’s urban GDP and 53.9% of its jobs; although informal businesses benefit urban populations immensely, insurance companies typically do not cover them.^19^

This initial survey study is a part of a larger randomized controlled trial of small business disaster microinsurance funded by the Humanitarian Innovation Fund. This study investigates the concerns noted above and looks at the potential for microinsurance in poor urban areas. In each surveyed region, we gauged knowledge of microinsurance and small business owners’ desire for programs. This initial study report contextualizes those findings and assesses the potential success of microinsurance programs.

## Methodology

The study was conducted in partnership between the All India Disaster Mitigation Institute (AIDMI), an NGO based in Gujarat, India and Stanford University in California, USA supported by the Humanitarian Innovation Fund. The study sites include urban slums in three disaster-prone cities of India: Puri in Odisha, Guwahati in Assam and Cuddalore in Tamil Nadu. The demand survey instrument was designed collaboratively between the two organizations with feedback from local community-based organizations (CBOs) in the study cities. After teams at AIDMI and Stanford had finalized the survey template, it was sent to CBO staff in the field.

To understand the effects of recent disasters on small business owners, local teams surveyed 1622 people in Guwahati, 1551 people in Puri, and 1746 people in Cuddalore from urban slum communities served by a local CBO in each region. These three pilot sites were selected because they met two important criteria: each lies in a high-risk zone for cyclones or flooding, and each had a community-based organization with the capacity and motivation to participate in this program. Purposive sampling was employed to identify small informal business owners to include and survey in the study. Since no comprehensive database of small business owners in slums exists, random sampling could not be conducted. Additionally, a purposive design enabled focus on the vulnerable vendors most in need of microinsurance programs. After obtaining consent, staff from each local CBO collected survey data from informal business owners at their shop on mobile platforms using the KoBo toolbox software, Cambridge, Massachusetts. The survey instrument included questions about demographics and household information. It also included questions regarding previous experience with disasters and gauged knowledge of and interest in disaster microinsurance. The data was compiled by AIDMI and analyzed at Stanford using Microsoft Excel, Seattle, Washington.

Ethical approval for this study was granted by the Human Subjects and Research Institutional Review Board at Stanford University, Protocol # IRB-33474.

## Results

Demographics from the three study sites are displayed in Table 1. In all three locations, males compose the majority of business owners. Most house owners had ownership documents, but many lived in kutcha houses — buildings made out of weak or flimsy materials — which would not fare as well in a natural disaster. Many also owned shops made out of temporary materials, defined as fragile due to their risk in the face of natural disasters. In Guwahati and Puri, around half of the small businesses were mobile, though many still set up and kept their business materials in hazardous areas.

Many vendors sold food and other goods; 12% to 31% sold fruits and vegetables. Others offered bread or other groceries. And a few offered sundry items or more specialized products. Notably, 26% of Puri’s vendors sold handicrafts (owing to Puri’s beach tourism), and 20% of Guwahati’s merchants sold pan, but few sold either in the other study sites.

Though income varied, on average, small business owners made between Rs. 7,000 and Rs. 9,000 each month.

**Figure:**
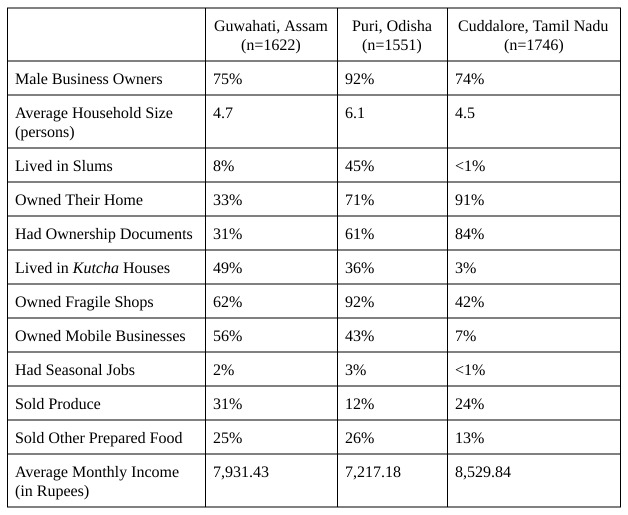
**Table 1**: Demographic Information

Experience with previous disasters is summarized in Table 2. Nearly everyone in all three sites had been affected by disasters at some point, and a considerable number had had their businesses or goods damaged. However, each community struggled with slightly different challenges. In Guwahati, 67% of people who reported being affected by disasters listed floods as being particularly damaging. And in both Puri and Cuddalore, everyone impacted by disasters referred to cyclones. People in Puri specifically complained about the Odisha Super Cyclone (1999), Cyclone Phailin (2013), and Cyclone Hudhud (2014).

Respondents reported varying levels of destruction. In Cuddalore, for example, 61% of those who had damaged businesses said they suffered major damage, and 34% had lost their businesses completely. In Guwahati, 23% of those with damaged businesses described major damage.

**Figure:**
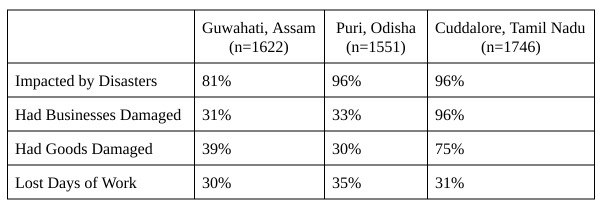
**Table 2**: Effects of Disasters

Prior to explaining microinsurance, surveyors asked vendors how they would respond to a natural disaster; the results are shown in Table 3. In Guwahati, 53% of respondents said that they would use savings to cope. Most people in Puri and Cuddalore — 72% and 100%, respectively — said they would take out a loan. Others noted they would sell their assets or rely on aid. However, all of these options can be detrimental to business owners and their families.

**Figure:**
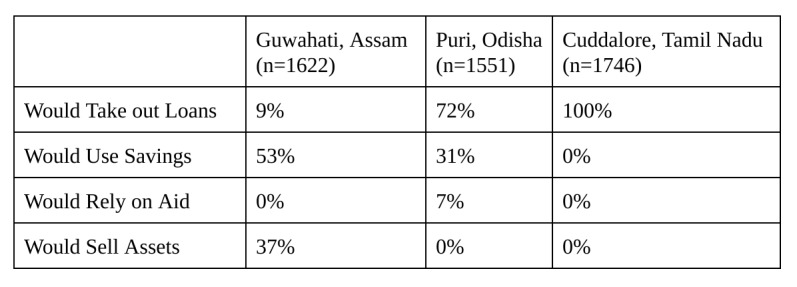
**Table 3**: Current Coping Mechanisms

To learn more about the potential for future programs in the region, surveyors specifically asked about knowledge of and demand for microinsurance, displayed in Table 4. While the numbers within each site varied, most business owners responded very favorably to the idea of microinsurance.

We found that, in general, wealthier households wanted to buy more expensive microinsurance plans, but the least expensive model (between 100 and 200 Rupees) suited most respondents. This held true across all three study sites.

The survey asked the respondents who did not want microinsurance to explain their aversion. By and large, of those who did not want insurance coverage in Guwahati and Puri — 44% and 71%, respectively — said they simply would not be able to afford the price. Because everyone interviewed in Cuddalore wanted disaster microinsurance, reasons for lack of demand cannot be reported. Some feared chit scams, a form of financial fraud that has taken advantage of people across India in recent years. Others did not believe that they faced enough risk to justify the purchase. Another group had purchased some form of insurance previously, but did not think that they needed it currently. However, the percentage of business owners not interested in microinsurance made up a small minority in all three study sites.

**Figure:**
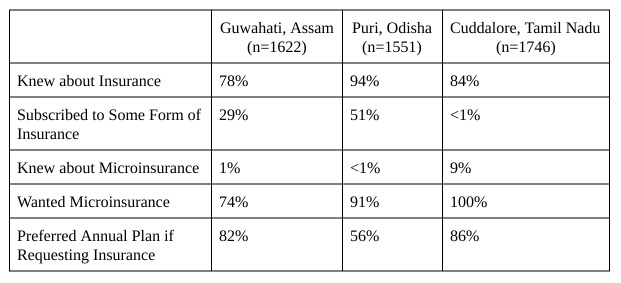
**Table 4**: Insurance and Microinsurance

**Figure:**
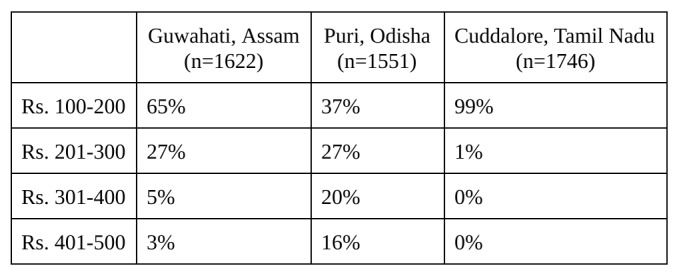
**Table 5**: Preferred Premium

**Figure:**
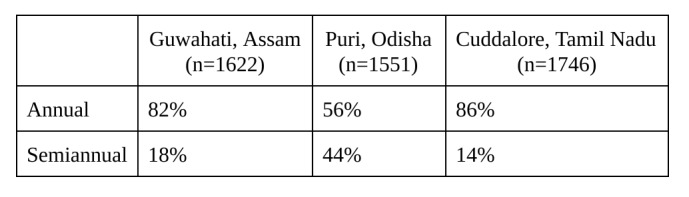
**Table 6**: Preferred Renewal Period

**Figure:**
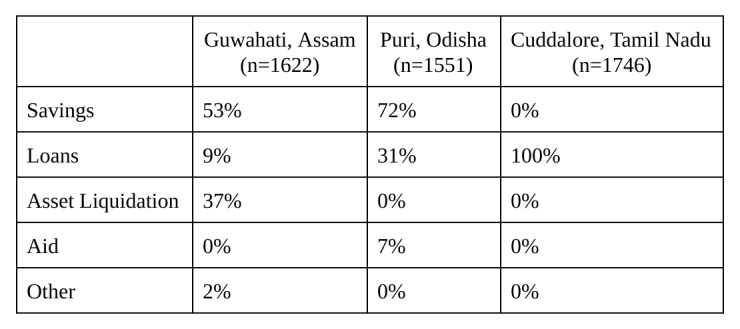
**Table 7**: Current Response to Disaster

**Figure:**
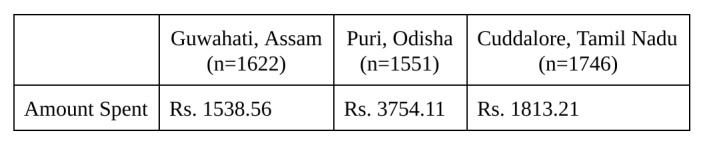
**Table 8**: Current Amount Spent on Recovery

**Figure:**
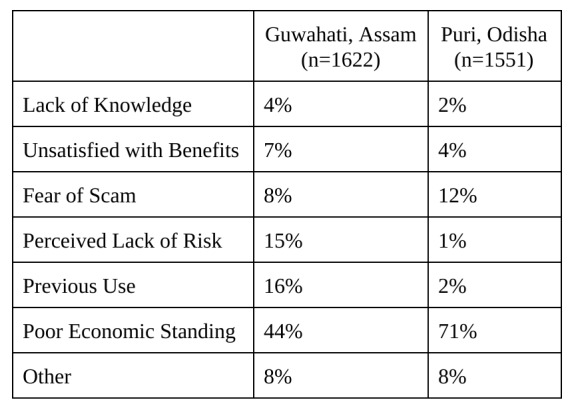
**Table 9**: Reasons for Rejection of Microinsurance

## Discussion

The comprehensive data from this initial survey of almost 5000 business owners shows that small informal business owners largely do not know about disaster microinsurance for businesses. The survey demonstrates that this lack of awareness pervades multiple regions of India — including high-risk zones.

The study also corroborates the hypothesis that small informal business owners have low awareness of microinsurance for businesses. The survey demonstrates that this lack of awareness exists across multiple regions of India, even in high-risk zones prone to frequent disasters.

But it also highlights the opportunity to create demand by raising awareness. The first operational goal for microinsurance providers entering this space should be to explain how risk-transfer programs function and benefit business owners; based on the high demand indicated by respondents following an explanation, this strategy seems viable. As a risk-pooling mechanism, the success of microinsurance programs depend on a robust and diverse user base. The data above clearly show that the majority of small business owners would purchase an affordable plan. Often, witnessing payouts makes microinsurance more attractive, meaning the subscriber base will likely grow after a disaster (furthering the financial viability of the program).

Beyond generating demand, insurance providers will have to design products and delivery methods that function and scale easily. Insurance requires an implicit level of trust. Unfortunately, many of these communities have been the victims of fraud, often in the form of chit schemes, where many individuals put money into a pot and a randomly selected winner receives the money. Chit schemes and other financial scams can easily be confounded with legitimate insurance. To combat this confusion, community-based organizations can institute partner-agent models, in which staff serve as insurance agents by signing up subscribers and remaining a point of contact in the community. This model overcomes the logistical challenges of serving urban slum populations that have been largely neglected by financial institutions. In addition, community-based organizations already have the social capital and history in the community through previous development projects, allowing them to work effectively. Finally, the cost of insurance and the price of covering high-risk areas can hinder adoption rates and scalability. However, this survey shows that even small business owners have the ability and inclination to afford a product that is priced right.

Our study indicates that small informal business owners have suffered extreme loss in recent disasters. They currently resort to various coping mechanisms in lieu of formal risk reduction and risk transfer options. Unfortunately, many business owners rely on unsuccessful or even counterproductive coping strategies that trap families in poverty. These alternative coping strategies, including taking out loans and selling assets, would be obviated or limited by a microinsurance product. Insurance companies should be excited by this opportunity; the findings of this study support the possibility of price discrimination in real-world programs. Most small business owners wanted to purchase the most comprehensive insurance product they could afford, suggesting an opportunity for a range of insurance coverage and pricing.

This paper looked specifically at knowledge of and demand for small business disaster microinsurance across three different regions in India. To create a more complete picture of the impacts, more research — as planned in a randomized controlled trial of disaster microinsurance for this population — is required. It is important to note that intent does not necessarily equal action; people will vote with their wallets. Feasibility studies remain pertinent, especially since cost was the main reason stated for not participating. Future surveys and trials will be needed to test actual demand for microinsurance and actual impact on post-disaster recovery.

Governments and nonprofit organizations are actively exploring ways to help small and informal business owners to facilitate economic development. Humanitarian efforts are also looking to leverage markets found in urban areas to speed recovery after crises. Yet despite years of existence, disaster insurance for businesses remains underdeveloped and underused as a means to protect the poor and vulnerable. However, it has the potential to fill the gaps in financial inclusion seen in various products and services. While further operational research is needed, this study demonstrates that risk transfer for small informal business in urban slums shows promise as a revolutionary product for populations at risk.

## Data Availability Statement

The data used to produce the tables above is freely available at Figshare via the following DOIs: https://doi.org/10.6084/m9.figshare.4632682.v1; https://doi.org/10.6084/m9.figshare.4632880.v1; https://doi.org/10.6084/m9.figshare.4632280.v1

## Competing Interests Statement

The authors have declared that no competing interests exist.

## Corresponding Author

Ronak B. Patel who may be contacted at rbpatel@gmail.com.
